# Fruit and vegetables loss and waste in preschools belonging to the National Board of Kindergartens of Chile

**DOI:** 10.23938/ASSN.1089

**Published:** 2024-10-14

**Authors:** Ximena Rodríguez Palleres, Claudio Villota Arcos, Álvaro Toledo San Martín, Fancy Rojas González, Juan Manuel Castagnini

**Affiliations:** 1 Universidad de Valencia Facultad de Farmacia Departamento de Medicina Preventiva y Salud Pública, Ciencias de la Alimentación, Toxicología y Medicina Forense. Grupo de Investigación en Tecnologías Innovadoras para una Alimentación Sostenible (ALISOST) Valencia España; 2 Bernardo O’Higgins University Faculty of Health Sciences School of Nutrition and Dietetics Santiago Chile; 3 Bernardo O’Higgins University Faculty of Engineering, Science and Technology Department of Mathematics and Engineering Sciences Santiago Chile; 4 Bernardo O’Higgins University Department of Transfer, Entrepreneurship and Innovation Santiago Chile

**Keywords:** Food Loss and Waste, Fruit, Vegetables, Schools, Nursery, Desperdicio de Alimentos, Fruta, Verduras, Jardines Infantiles

## Abstract

**Background:**

Food waste is a global issue affecting society from environmental, nutritional, and social perspectives. In collaboration with the National Board of Preschools, fruit and vegetable waste generated during the preparation of lunch was quantified in four preschools in the Metropolitan area of Santiago in Chile.

**Methods:**

This study was conducted in four preschools, two in the Western area of Santiago and two in the Eastern area, of which two hold environmental quality certifications. Over a five-day period, the weight of raw materials and waste from vegetables (peels) and fruit (pomace) were measured. Percentages of vegetable and fruit post-cleaning losses and waste were evaluated.

**Results:**

Vegetable loss exceeded 20% in 31% of the preschools, primarily from carrots and potatoes. Fruit losses were higher, with pears accounting for the most significant waste, recorded in 75% of the study centers. No differences in vegetable loss was found between centers, while variations were observed for fruit. Preschools with environmental quality certifications wasted less onions (p = 0.016) but more pears (p = 0.036).

**Conclusions:**

There is higher fruit loss than vegetable loss, with onions and tomatoes being the least wasted. Possessing an environmental quality certification does not guarantee a significant reduction in overall losses. Handling and storage conditions may play a key role in minimizing losses. Further studies are needed to provide evidence that can guide improvements in Chile’s National Board of Preschools services, aiming for a more sustainable lunch preparation processes.

## RESUMEN

**Fundamento:**

Los desperdicios de alimentos son un problema global y afectan a toda la sociedad desde una perspectiva ambiental, nutricional y social. En colaboración con la Junta Nacional de Jardines Infantiles, se cuantificó el desperdicio de frutas y verduras durante la elaboración de raciones en cuatro jardines infantiles de la Región Metropolitana de Santiago en Chile.

**Metodología:**

Se seleccionaron cuatro jardines infantiles, dos con sello de calidad ambiental. Durante cinco días se pesaron la materia prima y los desperdicios de verduras/hortalizas (peladuras) y frutas (pulpa), y se calculó el porcentaje de pérdida post limpieza.

**Resultados:**

Las pérdidas de verduras/hortalizas fueron > 20% en el 31% de los centros, especialmente en el caso de zanahorias y patatas; las pérdidas de fruta fueron superiores, especialmente en el caso de las peras, en el 75% de los centros. No hubo diferencias entre centros para pérdidas de verduras/hortalizas, pero sí para ambos tipos de fruta. Los centros con sello ambiental desperdiciaron menos cebollas (p = 0,016) y más peras (0,036).

**Conclusiones:**

Se observa mayor pérdida de fruta que de verduras/hortalizas, siendo las cebollas y los tomates los menos desperdiciados. Estar en posesión del sello medioambiental no se relaciona a una reducción significativa de pérdidas, excepto en el caso de las cebollas. La manipulación y las condiciones de conservación podrían ser determinantes para la reducción de desperdicios. Se necesitan más estudios que recolecten evidencia que permita proponer mejoras en los servicios de Junta Nacional de Jardines Infantiles para lograr un proceso de elaboración de raciones de comida más sostenibles.

## INTRODUCTION

Reducing food loss and waste is a great challenge of modern times in developed countries. Public and private institutions are focusing their efforts on reducing hunger, increasing incomes, and improving food security in lower-income countries^1^. Official figures indicate that approximately a third of all food produced for human consumption in the world is lost or wasted along the food supply chain each year^2^, with an economic impact of over 1.3 billion dollars every year^3^. Vegetables and fruits account for 40% to 50% of food loss and waste, 39% of the waste occurring in homes^4^. Food loss affects local and global economy and generates waste of resources used in their production such as lands, water resources, energy, inputs, and CO_2_ emissions^5^.

The term *food loss* is defined as the reduction in the amount of edible food that is available for consumption^6^. Food loss can occur at any stage of the food production chain (production, post-harvest, storage, or processing stage) - before reaching the market - that is not fit for human consumption. The stages where losses can be most easily addressed are storage and processing. Factors contributing to food loss include food spoilage, sprouting, and dehydration, frequently caused by poor handling or inadequate facilities. The processing of fruits and vegetables is often associated with losses due to improper handling and the removal of unsuitable or contaminated products^7^. These food losses are higher in developing countries. FL undermine the sustainability of food systems, reduce food availability and access, and lead to inefficient use of the resources. Additionally, significant nutritional losses occur in foods with high nutritional value, such as vegetables and fruits, which are the most wasted^8^.

*Food waste* is defined as the loss that occurs at delivery points (markets, warehouses, or fairs) or due to poor management in homes, food stores, and restaurants^5^. At home, the primary causes are excessive food purchasing and poor food selection^9^.

Although Chile is a major producer of fruits and vegetables, it has not succeeded in reducing food insecurity and the rates of food loss^7^. The most commonly consumed fruits and vegetables in Chile include carrots, tomatoes, pumpkins, and apples. Since these foods are typically peeled before consumption, they contribute to food loss percentages of around 20%, 10%, and 8%, respectively^10^. For instance, potato peels account for 2% of this vegetable^11^. Depending on the used peeling technique, potato peel waste can vary between 15% and 40%^12^. There is a significant body of literature on food loss and food waste that, in Chile, are recognized as a national issue. But, to the best of our knowledge, no studies have been conducted in the country that quantify food loss during food preparation processes in educational establishments such as preschools or nurseries. At the national level, studies have examined the acceptability of the School Feeding Program (PAE), as well as children’s food preferences and the overall impact of the program.

Two governmental entities in Chile ensure the well-being of the children/adolescents from pre-school through university: the National School Aid and Scholarship Board (*JUNAEB*) and the National Preschool Board (*JUNJI*). Both institutions have taken the challenge of promoting healthy eating and evaluated its impact on the population^13^. The missions of the *JUNJI,* established in 1970, is to provide quality early childhood education and promote comprehensive well-being for children and families facing greater socioeconomic vulnerability. The *JUNJI* operates a network of preschools across the country, providing education and meals for the children. Most public preschools in Chile are part of the *JUNJI* network. These settings use state resources to provide healthy meals for children under three months of age (84 days) and up to four years (3 years and 11 months).

In 2021, a study on the food preferences of schoolchildren in the Coquimbo Region revealed a low acceptability of vegetables and legumes, alongside a strong preference for beef consumption^14^. Beef is not used preferentially in JUNJI meal plans, as the focus is on promoting the consumption of lean meats or those with better nutritional quality, such as fish and chicken.

The latest evaluation report on the impact of JUNAEB policies^13^ shows a positive effect on meeting nutritional requirements and expanding the coverage of the PAE program, among other outcomes. The report shows that 41% of the beneficiary students like the food, 28% consider it good or very good, and 15% rate it bad or very bad. To date, there has been no assessment of consumption preferences among beneficiaries of the JUNJI. This is the first study of its kind developed in Chilean preschools.

The aim of this study was to evaluate the percentage of vegetable peel and fruit pomace loss during food preparation in JUNJI preschools. Identifying the causes of this waste will provide valuable data for developing new protocols to reduce fruit and vegetables waste.

## MATERIALS AND METHODS

Cross-sectional, observational study conducted in four preschools of the Metropolitan area of Chile, intentionally and non-randomly selected, between the months of March and June 2023.

**Figure 1:**
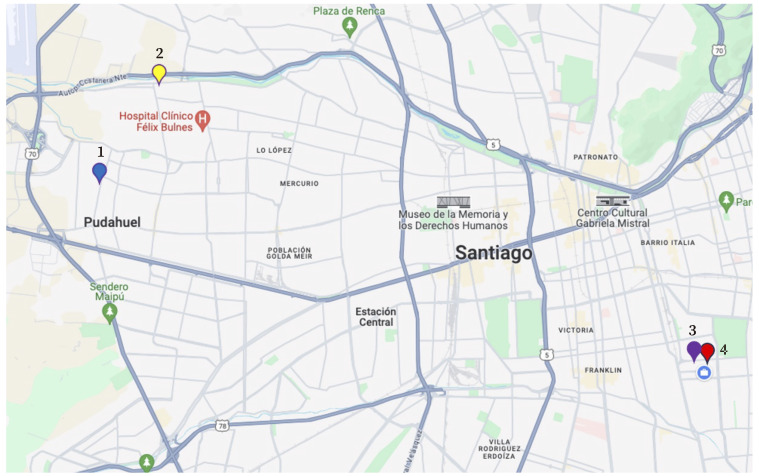
Location of the four preschools included in the study within the Metropolitan Area of Chile. Preschools 3 and 4 hold an environmental quality certification.

Two of the four centers evaluated (Preschools 1 and 2) are located in the Western area of the Metro-politan area of Chile and do not have environmental quality certifications, while preschools 3 and 4 correspond are in the Eastern area (Fig. 1) and hold environmental quality certifications. The environmental quality certification reflects the commitment of the preschools to reduce their environmental impact through activities such as composting, creation of gardens, recycling, and incorporation of eco-friendly educational practices in the preschools.

In Chile, all preschoolers participate in a free feeding program that provides breakfast (09:00), lunch (11:00-12:30), and an afternoon snack, known as *la once* (15:00-16:00). The afternoon snack often includes bread with less healthy additions such as cheese or sausages.

The main fruit and vegetable waste was assessed over a 5-day period. In preschools 1 and 3 data were collected over five consecutive days from Monday to Friday, while in preschools 2 and 4 data collection occurred from Monday to Thursday, with the fifth day postponed to the following Monday, due to administrative scheduling issues. The weight of the five primary fruits and vegetables used (potatoes, carrots, onions, tomatoes, apples, and pears) was measured both before and after lunch preparation, mainly during the peeling and cleaning stages. A nutritionist performed the weighing of food and food waste using electronic kitchen scales (Camry EK3252, China) and recorded the data in an Excel spreadsheet.

This study was approved by the Ethics Review Committee of the Faculty of Health Sciences of the Bernardo O’Higgins University (Santiago, Chile), resolution CFACS072022.

The data are presented as the mean percentage of loss for each preschool, along with the standard deviation (SD). The normality of data was assessed using the Shapiro-Wilk test, and homogeneity of variances was evaluated with Levene’s test. To determine significant differences in the mean percentage of loss for each meal and preschool, data were analyzed using the Kruskal-Wallis test, followed by pairwise comparisons (Mann-Whitney U with correction for multiple comparisons). Results were compared based on the presence of an environmental quality certification using the Student’s-t test (for vegetables) and the Mann-Whitney U test (for fruits). Statistical analyses were performed using the RStudio software (version 2023.03.0+386).

## RESULTS

Losses ranged between 8% and 20% for 68.75% of the vegetables. The highest losses were observed for potatoes (19% to 24%) and carrot peels (21% to 26%, except for preschool 2), while the lowest losses were found for onion peels (< 10%) and tomato pomace (< 13%) (Table 1). Although the data showed homogeneity of variances, they did not follow a normal distribution; therefore, the Kruskal-Wallis test was applied, revealing no statistically significant differences among the preschools.

**Table 1:** Vegetables losses in the National Preschool Board preschools in the Metropolitan area of Chile

Vegetables losses	Loss [mean % (SD)] by preschool				p-value Kruskal-Wallis
	1	2	3	4	
Carrot peel	21.59 (7.79)	11.10 (0.41)	25.31 (15.02)	26.03 (11.28)	0.155
Onion peel	9.42 (0.25)	9.59 (0.42)	7.99 (1.55)	8.23 (1.24)	0.055
Potato peel	23.99 (0.40)	19.59 (0.96)	20.00 (0.02)	21.69 (2.59)	0.234
Tomato pomace	12.05 (2.11)	9.55 (0.30)	10.49 (5.35)	12.39 (4.29)	0.927

SD: standard deviation.

For fruits, 75% of the losses ranged from 30% to 82%, with the highest losses attributed to pear pomace. Only in preschools 1 and 4 were losses below 20%, both related to apple pomace. The highest loss was seen for pear pomace in Preschool 3, while the lowest was for apple pomace in preschool 1 (Table 2). Significant differences were found between preschools for both fruits (Kruskal-Wallis test). According to *post hoc* tests, apple pomace loss was higher in preschool 3 compared to the other preschools, and lower in preschool 2 compared to preschool 4. Mean apple pomace losses in preschools 1 and 4 were very low compared with that of preschool 3, although significant differences were only observed between preschools 2 and 3 in the *post hoc* analysis).

**Table 2:** Fruit losses in the National Preschool Board facilities in the Metropolitan Area of Chile

Fruit losses	Loss [mean % (SD)] by preschool				p-value Kruskal-Wallis
	1	2	3	4	
Apple pomace	2.82 (5.66)	34.17 (35.39)	72.48 (32.22)	7.72 (13.50)	< 0.001
Pear pomace	37.15 (33.06)	29.97 (35.28)	82.36 (16.39)	39.26 (53.77)	0.036

SD: standard deviation.

Comparing vegetable loss percentages between preschools with and without environmental quality certifications, the highest loss in certified preschools was for potato peels, while in non-certified preschools, it was from carrot peels. Potato and tomato losses were similar regardless of certification, but certified preschools had significantly lower losses of onion peels (p=0.016) and higher, though not statistically significant, losses of carrot peels. Preschools with environmental quality certification had higher fruit losses, with pear pomace loss being double the percentage compared to non-certified preschools (Table 3).

**Table 3:** Food losses in National Preschool Board in Chile facilities in the Metropolitan Area of Chile based on environmental quality certifications

Food losses	Loss [mean % (SD)] by preschool		p-value
	Without EQC (1/2)	With EQC (3/4)	
Vegetables			Student’s-t test
Carrot peels	15.59 (7.19)	25.76 (11.72)	0.069
Onion peels	9.47 (0.28)	8.15 (1.19)	0.016
Potato peels	20.42 (1.96)	21.21 (2.27)	0.516
Tomato pomace	11.22 (2.09)	11.77 (4.21)	0.781
Fruits			Mann-Whitney U test
Apple pomace	30.34 (34.74)	40.11 (41.01)	0.598
Pear pomace	30.56 (34.89)	60.81 (42.82)	0.036

EQC: Environmental Quality Certification; SD: standard deviation.

Preschools 1 and 2, located in the Western area, had lower percentages of food waste compared to Preschools 3 and 4, located in the Eastern area and with environmental quality certifications. Although no significant differences were found between the four preschools (except for pear pomace), statistical differences were observed between certified and non-certified preschools for onion peels and pear pomace. Moreover, differences were noted between preschools 3 and 4 both located in the Eastern area and holding environmental quality certification.

## DISCUSSION

To the best of our knowledge, this study is the first to assess the waste generated during lunch preparation in four JUNJI preschools in Chile, enabling the development of new protocols for managing food waste.

The results show that some food losses are comparable to those reported in other studies, ranging from 10% to 20%, depending on the type of fruit or vegetable^10^; however, notably high losses are observed for carrots, potatoes, apples, and tomatoes. These figures are not surprising, as fresh vegetables and fruits are among the most wasted food groups. Additionally, in retail, food services, and households, losses of fruits and vegetables account for 22% of total waste^15^.

In this study, losses for carrots (20%; only preschool 2 was below this), potato peels (15-20%; all preschools were at or above the upper end), and apples (8%; all preschools exceeded 9%) are higher than those reported in the literature^10^.

The environmental quality certification is linked to recycling and composting at the preschools. Our results indicate that there is no reduction of food waste in preschools with this certificate, except for onion peels. In this study, waste designated for composting is not counted as waste; only the weight of the food and its corresponding peels and pomace were measured. Consequently, food waste may be attributed to the fact that children in the Eastern area have different food preferences and do not consume the same foods as their peers in the Western area.

Given that a common cause of food waste is the condition of fruits and vegetables^6^, it is interesting to compare the results from preschools 3 and 4, both located in the Eastern area and holding an environmental quality certification. Furthermore, since they use the same supplier for fruits and vegetables, it is likely that the differences described above arise during the handling of these products. The handling practices or knowledge of food handlers has not been evaluated, so this variable cannot be confirmed or ruled out. If losses are attributed to handling rather than to the quality of the food, implementing changes such as training, adapting new spaces, or acquiring new kitchen tools, may help reduce these losses.

Just as fruits and vegetables contain bioactive nutrients, their peels are also valuable for their functional components, such as carotenoids, dietary fiber, polyphenols, and flavonoids^16^. Therefore, it is possible to extract high-value bioactive compounds and develop new foods through the valorization of fruits and vegetables, contributing to circular economy^17^^,^^18^. Among children, greater consumption of fruits and vegetables is associated with a lower risk of obesity, diabetes, hypertension, and high cholesterol, as well as the prevention of nutrient deficiency. Therefore, promoting adequate intake of these products, along with addressing any surplus, is of utmost importance^19^.

In this study, fruit and vegetable losses range from 2.82% to 82.36%, which is largely consistent with data reported elsewhere^10^. This variation may be attributed to several factors, including inadequate cleaning processes, poor peeling techniques, substandard knives and peelers, and lower-quality raw materials supplied with higher degree of ripeness and mechanical damage. These factors ultimately reduce the quality of fruits and vegetables, leading to increased losses.

Undoubtedly, the involvement of all stakeholders, including preschool directors, nutritional advisors, concessionaires and suppliers of raw materials play a key role in reducing waste.

One of the strengths of this study is its methodology. Direct weighing to quantify fruit and vegetable waste is a simple, reproducible method that can also be applied to other settings such as schools, universities, hospitals, and clinics. All fruit and vegetable waste measurements were performed by nutritionists with knowledge and experience in this type of data collection, and the researchers made multiple visits to the preschools during the waste measurement process to promptly identify and address any issues. The main limitations to this study are the small number of preschools assessed and the fact that all are located in the Metropolitan area of Chile. However, their selection, based on the presence of environmental quality certification and geographical location, allowed for a detailed analysis and comparison of the results. Another limitation is the lack of up-to-date scientific evidence on food losses during the preparation of meals, which would have allowed for comparisons with other national and international contexts.

In conclusion, vegetable losses range between 8% and 26.03%, while fruit losses reach up to 82.36%, with the highest losses occurring in potatoes and pears. There is no significant reduction in vegetable losses in preschools with an environmental quality certificate, except for onion peels, and the average loss of pear pomace doubled that of preschools without a certificate. Thus, other factors, such as handling, processing techniques, and conservation conditions, may play a more decisive role in reducing waste. The quality of fruits and vegetables supplied, combined with management and cleaning practices in the preschools, influence the variability of losses. Interventions targeting these areas could significantly reduce waste.

Larger-scale studies should be conducted to develop more effective management protocols and adapt contracts with suppliers. This would improve current conditions, reduce food waste, and maximize the resources at the JUNJI preschools, ultimately leading to a lower environmental impact.

## Data Availability

Data are available under request to corresponding author.
